# Metastatic patellar bone tumor due to gastric cancer resembling a primary or secondary aneurysmal bone cyst: A case report

**DOI:** 10.1016/j.ijscr.2023.108379

**Published:** 2023-06-02

**Authors:** T. Furuta, T. Sakuda, K. Yoshioka, K. Arihiro, N. Adachi

**Affiliations:** a Department of Orthopaedic Surgery, Hiroshima University, Graduate School of Biomedical and Health Sciences, Hiroshima, Japan; b Department of Pathology, Hiroshima University Hospital, Hiroshima, Japan

**Keywords:** Metastatic bone tumor, Patella, Patellectomy, Gastric cancer

## Abstract

**Introduction and importance:**

Patellar bone tumors are very rare, and most are benign or of intermediate type. In this report, we describe our experience of a metastatic patellar bone tumor caused by gastric cancer, which resembled a very rare primary or secondary aneurysmal bone cyst and review the literature.

**Case presentation:**

A 65-year-old man presented with severe pain in the patellar region and marked limitation of the knee joint range of motion. He had a history of gastric cancer; however, epidemiological, clinical, and imaging findings led us to strongly suspect an aneurysm-like bone cyst. Thus, we performed bone tumor curettage and autologous artificial bone grafting without biopsy because of the severe pain. Pathology results showed gastric cancer metastasis; hence, patellectomy and patellar tendon augmentation with femoral fascia were performed. The Musculoskeletal Tumor Society (MSTS) score was taken postoperatively to assess pain and function.

**Clinical discussion:**

We experienced a very rare gastric cancer-related metastatic patellar bone tumor, which resembled a primary or secondary aneurysmal bone cyst in frequency and imaging findings. Patellectomy was ultimately performed, and the patient's MSTS score improved markedly.

**Conclusion:**

Despite its very low frequency, patellar metastatic bone tumors must be taken into account without being misled by the frequency or imaging findings and a biopsy should necessarily be performed.

## Highlights

• Patellar bone tumors are extremely rare, and metastatic bone tumors of the patella are even rarer.
• Experienced patella gastric cancer bone metastasis that has not yet been reported
• Patellectomy for metastatic bone tumor satisfied patient
• Infrequent patellofemoral tumors should be biopsied and a treatment plan should be developed.

## 1 Introduction

Among primary bone tumors, patellar tumors are very rare, accounting for <1 % of all bone tumors. Most patellar tumors are benign or intermediate tumors, and giant cell tumors of bone and chondroblastoma are the most commonly reported [[1], [2], [3]]. Others such as osteoid osteomas, intraosseous lipomas, and intraosseous hemangiomas have also been reported [[4], [5], [6]]. Conversely, metastatic patellar tumors are infrequently reported, with only a few case reports, and primary sites include the lung, kidney, esophageal, malignant melanoma, colon, and head and neck cancer [[7], [8], [9], [10], [11]].

To our knowledge, no study has reported metastatic bone tumors in gastric cancer. On the other hand, aneurysmal bone cyst is a benign blood‑tinged cystic neoplasm of bone. It may present as a primary tumor or may be associated with other neoplastic disease such as giant cell tumor of bone or metastatic bone tumor [12].

Based on our reflections throughout this case, we discuss herein the appropriate treatment strategy for patellofemoral tumors and review relevant literature.

We gave the patient informed consent to publish the article and he readily consented. Treatment interventions were performed in compliance with SCARE 2020 [14].

## 2 Presentation of case

**Fig. 1:**
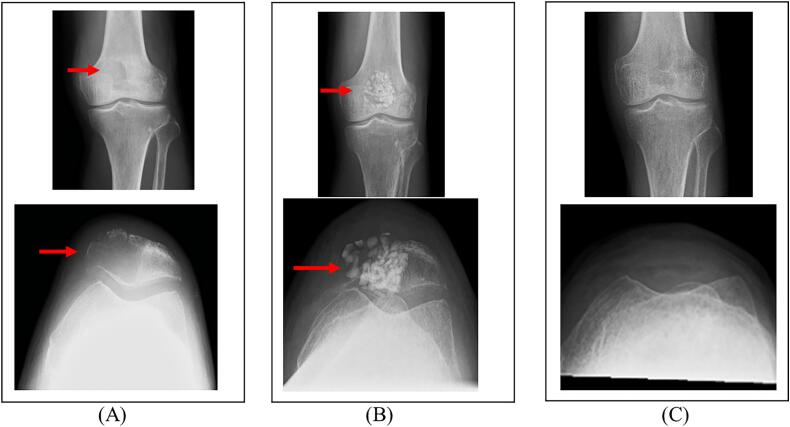
Preoperative and postoperative radiographs of the left knee of a 65-year-old man. (A) Preoperative frontal and lateral views of the patella showing osteolysis (arrowhead). (B) Frontal and lateral views after curettage of the tumor and grafting of the artificial and autologous bones (arrowhead). (C) Frontal and lateral views after patellectomy.

**Fig. 2:**
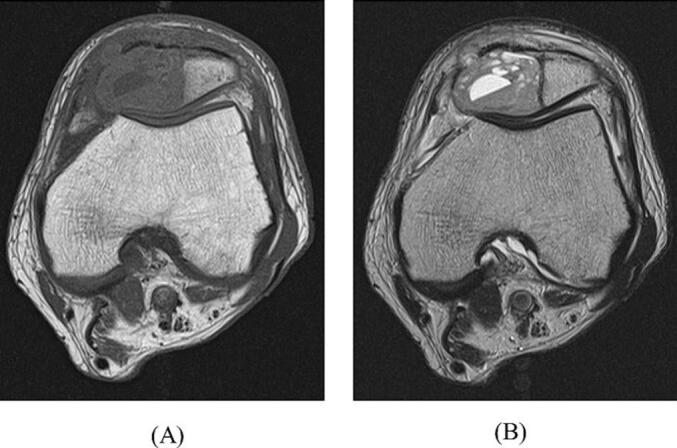
Preoperative images of a patellar tumor of the left knee. Magnetic resonance image of the left knee in the axial view. (A) T1-weighted image showing a lesion occupying the medial side of the patellar bone marrow. (B) T2-weighted image showing multifocal fluid formation within the patellar bone marrow, with some tumors infiltrating outside the patella.

A 65-year-old Japanese man who had a history of postoperative gastric cancer, had no psychiatric disorder, and was taking analgesics presented with marked pain and swelling of the left knee for 2 months duration. He was suspected of having a patellar tumor based on X-ray examination and magnetic resonance imaging (MRI) by his previous physician and was referred to the Department of Bone and Soft Tissue Oncology of our hospital. Physical examination revealed marked tenderness in the patellar region and limited left knee joint flexion. His MSTS score was 1/30 (3.3 %). Radiographs showed osteolysis of the left medial half of the patella (Fig. 1A). MRI showed a patellar bone tumor with T1 isointensity and heterogeneous high and very high T2 intensity. Moreover, multifocal fluid–fluid level formation was observed. The tumor destroyed the medial patellar bone cortex but had not invaded beyond the suprapatellar membrane (Fig. 2).

**Fig. 3:**
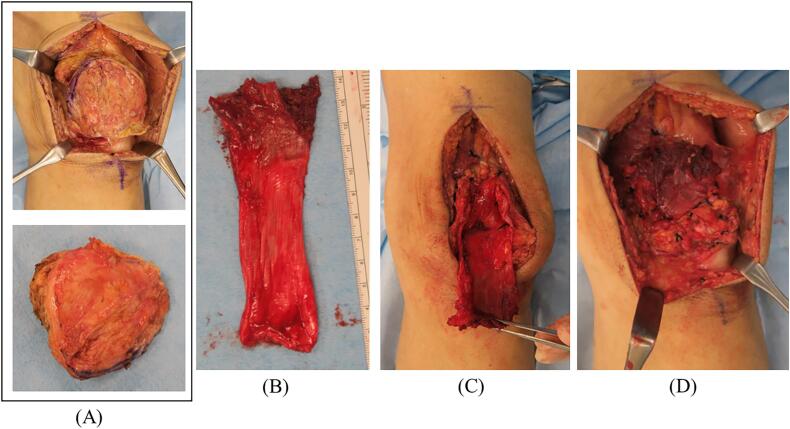
Preoperative images of the malignant scapular tumor in the right shoulder. On magnetic resonance imaging, the frontal view of the right shoulder (A) showed malignant tumor invasion of the entire scapula and glenoid fossa but not the acromioclavicular joint. The axial view of the right shoulder (B) showed malignant tumor invasion of the deltoid muscle (abductor muscle). Three-dimensional computed tomographic angiography (C) showed several feeding vessels extending from the axillary artery to the malignant tumor.

**Fig. 4:**
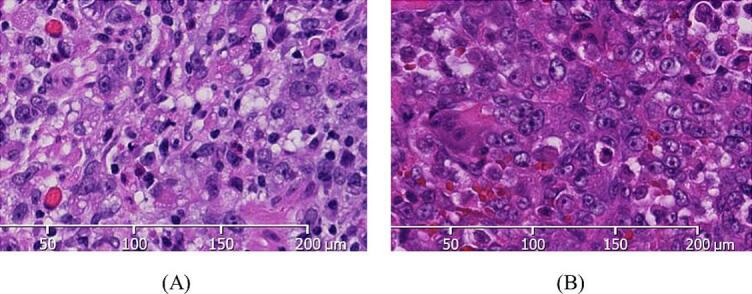
Intraoperative photographs. (A) The Malawer technique type IVB was used to remove the entire scapula, proximal humerus, and part of the abductor muscles, but the clavicle was preserved. (B) The tumor was widely resected. (C) The head of the proximal humeral prosthesis was placed at an inverted 180° angle to avoid pressure on the axillary artery and brachial plexus in contrast to the instructions in the product manual. Nesplon tape was fixed tightly to the clavicle and the clavicle-locking plate so that the tape was sandwiched between them. (D) A clavicle-locking plate, Nesplon tape, and a proximal humeral prosthesis were used to achieve shoulder stability.

The patient had a history of gastric cancer, and metastatic bone tumor was considered a differential diagnosis. However, metastatic bone tumors of the patella are extremely rare. First, a primary or secondary aneurysmal bone cyst was the most likely diagnosis based on the imaging findings, and second, the patient was in great pain. Even more rigid splint fixation did not improve the pain. A needle biopsy or biopsy was recommended first, but the patient did not agree, insisted that he could not wait for the biopsy results, and strongly preferred surgery; thus, the first stage of excision was performed without an incisional biopsy. Because the surgery was a semi-emergency surgery, a rapid pathology examination could not be performed at night because the pathologist was not present. All surgeries were performed entirely by the author, who has specialized in bone and soft tissue tumors for 12 years. A skin incision was made just above the patella to expose the suprapatellar membrane. A longitudinal incision was made through the suprapatellar membrane to expose the patella. The superior margin of the medial half of the patellar cortex was osteolyzed. The area was scraped firmly with an Air Speed Bar to ensure that no tumorous tissues remained. Anhydrous ethanol treatment was repeated six times for 3 min in the patellar medulla. Bone grafting was performed by mixing the bone taken from the left iliac bone and granular artificial bone and the femoral fascia seals the grafted bone to achieve stability, and the surgery was completed (Fig. 1B). Postoperatively, the patient was not in pain, was able to walk, and was doing well. However, 2 weeks later, the pathology results indicated metastasis of gastric cancer. A full-body computed tomography (CT) was immediately performed, which confirmed the absence of metastatic findings. An anhydrous ethanol treatment was initiated, but given the recurrence risk, a patellectomy was performed. The suprasellar membrane and patella were resected so that the tumor was not exposed. The deficient patellar tendon area was reinforced by taking a 12 cm × 6 cm piece of the fascia of the tensor fascia femoris muscle and placing it over two pieces (Fig. 3A–D). X-rays after patellectomy were unremarkable (Fig. 1B). Postoperative pathology revealed a residual tumor in the bone marrow, but the resection margins were negative. At the time of the previous gastric cancer surgery, the pathology showed the growth of substantial cells that did not retain the glandular duct structure, and the pathology of the poorly differentiated adenocarcinoma and the present patellar bone tumor matched (Fig. 4A, B). The patient was immobilized with a knee brace for 2 weeks postoperatively, after which range of motion training was started, and the patient could walk freely 4 weeks postoperatively. Six months after surgery, the bone tumor had not recurred, and the patient had not experienced difficulty in daily life.

The final MSTS score was 26 (86.6 %), and the left lower limb function was markedly improved [13]. Twelve months postoperatively, the patient's condition was improving without metastasis and without difficulty in daily living. Patients were very satisfied with the treatment results.

## 3 Discussion

Bone tumors arising in the patella are extremely rare [[1], [2], [3]]; however, the gold standard treatment strategy is to base treatment decisions on the pathology results of biopsy [15]. This patient had a history of cancer and a biopsy was necessary. At the very least, we should have performed a rapid pathology to determine whether the disease was benign or malignant. There are four points we must reflect on.

First, frequency was considered. Bone tumors are extremely rare, accounting for <1 % of all tumors, and most are benign or intermediate tumors such as chondroblastomas, osteoid osteomas, aneurysmal bone cysts, and giant cell tumors of the bone [[1], [2], [3]]. Malignant tumors such as osteosarcomas, chondrosarcomas, and Ewing's sarcomas have also been reported, but are extremely rare [[17], [18], [19]]. Metastatic bone tumors rarely occur. There have been reports of metastatic bone tumors from lung cancer, renal cancer, esophageal cancer, colorectal cancer, malignant lymphoma, and malignant melanoma; however, to our knowledge, no study has reported bone tumors as metastasis of gastric cancer [[7], [8], [9], [10], [11]]. Therefore, we thought that patellar bone metastasis was unlikely. However, in this case, we found that we had to perform an unnecessary surgery in case of malignancy, and we believe that it is important to base the treatment strategy for bone tumors on the pathological diagnosis by biopsy.

Second, we contacted the general surgeon at the hospital where the gastric cancer surgery was performed, they indicated that this patient was being followed up regularly and that it was extremely unlikely that the cancer would metastasize. Therefore, we neglected to have a conference with the surgeon and pathologist at our hospital. We should have coordinated with other department in our hospital.

Third, the imaging findings showed osteolysis on radiographs and multifocal fluid -fluid level formation on T2 MRI, which, along with the frequency, led us to assume that it was an aneurysmal bone cyst. Therefore, we neglected to perform additional contrast-enhanced MRI and CT scans. We needed to consider the possibility of a secondary aneurismal bone cyst as well as primary aneurysmal bone cyst. Secondary aneurysmal bone cyst-like change can be associated with giant cell tumor of bone or contain metastatic tumors [12].

Forth, the patient was in severe pain. Despite rigid splint fixation, the pain relief was poor. The patient could not sleep because of intense pain. We explained to the patient the need for the biopsy, but he could not give his consent because he could not tolerate the period of waiting for the biopsy results. In addition, because it was a semi-emergency surgery and the pathologist was not available at night, a rapid pathology examination could not be performed. We believe that it was important to provide the patient not only with symptoms but also with psychological care to better understand the benefits of biopsy. We have treated the patient with no evidence. In short, this case highlights the importance of using biopsy to determine a treatment plan based on pathology results. However, even if biopsy in excisional biopsy because of the lack of substantial components and sampling errors that can occur. Furthermore, there is one further reflection on the surgical method. We determined grossly that it was an aneurysmal bone cyst, and after adequate scraping and alcohol treatment, an autologous artificial bone grafting was performed. However, as excisional biopsies cement filling would have been sufficient, given that the tumor might be malignant. Considering that the patient's iliac bone was sacrificed, the importance of biopsy was still highlighted here. For a partial continuity defect of the patellar tendon following patellar resection, and the patellar tendon defect was small at the time of resection and was reinforced with a double layer of femoropopliteal tensor fascia (Fig. 3B-D). A tendon graft was not necessary as reported [17,19]. Furthermore, in our country, allograft is challenging [16]. Reinforcement with fascia grafts was reasonable for small defects. Pathology results showed negative resection margins, symptomatic improvement, MSTS score of 26 (86.6 %), and absence of recurrence or metastasis during the short follow-up period of 10 months, As a result, patient satisfaction was achieved.

We experienced an extremely rare gastric cancer-related metastatic patellar bone tumor that resembled a primary and secondary aneurysmal bone cyst in frequency and imaging findings. We considered biopsy, but the patient did not give consent and we operated without biopsy because of the patient's symptom relief and the availability of a surgical slot. Biopsy should have been performed even for patellar bone tumor of rare malignancy as the gold standard treatment strategy because it would have required unnecessary surgery and led to dissemination.

## 4 Conclusion

We experienced a very rare gastric cancer-related metastatic patellar bone tumor that resembled a primary or secondary aneurysmal bone cyst based on frequency and imaging findings. With a history of cancer, rare metastatic bone tumors, even in the infrequent patella, must be considered and we should have been persuaded the patient to do a biopsy.

## Ethical approval

No ethical approval was necessary for the treatment or investigation of this patient.

## Author contribution

Taisuke Furuta contributed to the research concept and design, data collection, analysis and interpretation, oversight and leadership responsibility for planning and execution of research activities, and external mentoring of the core team. Tomohiko Sakuda contributed to the data collection of the study. Koki Yoshioka contributed to the data collection, research concept, and treatment design of the study. Koji Arihiro contributed to pathological diagnosis and data collection. Nobuo Adachi contributed to the concept, supervision, and guidance of the study.

## Research registration

Not required for this report.

## Provenance and peer review

Not commissioned, externally peer-reviewed.

## Consent

Written informed consent was obtained from the patient for publication of this case report and accompanying images. A copy of the written consent is available for review by the Editor-in-Chief of this journal on request.

## Declaration of competing interest

The authors declare no conflicts of interest.
